# Application of oxycodone in anesthesia induction and overall management of Da Vinci robot-assisted nephrectomy: A randomized controlled trial

**DOI:** 10.1097/MD.0000000000029893

**Published:** 2022-08-12

**Authors:** Haihong Wang, Yuanli Qiu, Qiang Zheng, Yijiao Chen, Liang Ma

**Affiliations:** a Department of Anesthesiology, Sir Run Run Shaw Hospital, School of Medicine, Zhejiang University, Hangzhou, China; b Department of Anesthesiology, Shaoxing Municipal Hospital, Shaoxing, China; c Department of General surgery, Sir Run Run Shaw Hospital and the Institute of Minimally Invasive Surgery, School of Medicine, Zhejiang University, Hangzhou, China; d Department of Urology, Sir Run Run Shaw Hospital and the Institute of Minimally Invasive Surgery, School of Medicine, Zhejiang University, Hangzhou, China.

**Keywords:** anesthesia, Da Vinci robot-assisted nephrectomy, oxycodone

## Abstract

**Background:**

This study aimed to evaluate the application of oxycodone in anesthesia induction and overall management of Da Vinci robot-assisted nephrectomy.

**Methods:**

A total of 42 patients undergoing Da Vinci robot-assisted nephrectomy with general anesthesia were selected. They were randomly divided into 2 groups: patients were induced with oxycodone (oxycodone group) and were induced with sufentanil (sufentanil group). The vital signs at the following time points were recorded: after injection of induced drugs (T1), during glottis exposure (T2), immediately after intubation (T3), 1 minute (T4), 2 minute (T5), 3 minute (T6), 5 minute (T7), 10 minute (T8) after intubation, during skin incision (T9), at the end of suturing skin (T10), during extubation (T11), and during hemodynamic fluctuations intraoperatively (T`). Numerical rating scale, facial affective scale and monitoring of adverse events (visual analogue scale, NVAS) were evaluated postoperatively at 1 hour (T’“1), 3 hours (T”“2), 6 hours (T”“3), 12 hours (T”“4), 24 hours (T”“5), and 48 hours (T”‘6).

**Results:**

The systolic blood pressure, the diastolic blood pressure and the mean arterial blood pressure showed no statistically different changes between the 2 groups. There were no statistical differences in heart rate and respiratory rate among various timepoints intraoperatively. There were statistical differences in Bispectral index (BIS) scores in T6 between the 2 groups. The numerical rating scale and facial affective scale scores were significantly lower in oxycodone group. Anesthetized with oxycodone, the pain did not affect the sleep of patients after operation. Also, the postoperative QoR-40 scores were lower in oxycodone group.

**Conclusion:**

Compared with sufentanil, anesthesia induction with 0.3 mg/kg oxycodone in Da Vinci robot-assisted nephrectomy can achieve mild pain and mild adverse responses in patients postoperatively.

## 1. Introduction

The Da Vinci surgical robot is currently the most widely used surgical robotic system worldwide.^[1]^ Compared with traditional laparoscopic partial nephrectomy, Da Vinci robot-assisted nephrectomy has potential in increased mechanical arms freedom, higher definition of 3D stereoscopic vision, faster postoperative renal function recovery, shorter thermal ischemia time and shorter renal wound suture time.^[2]^ Despite its high cost, Da Vinci robot-assisted nephrectomy has obvious advantages for renal carcinoma (diameter <4 cm).^[3]^

Da Vinci robot-assisted nephrectomy has a longer surgery time and pneumoperitoneum time, which requires high level management of intraoperative anesthesia.^[3]^ However, as the same with laparoscopic partial nephrectomy, Da Vinci robot-assisted nephrectomy usually uses sevoflurane-remifentanil(sufentanil)-based anesthesia.^[4]^ Simplified anesthesia has positive significances for emergency treatment and postoperative recovery of patients. Of these, oxycodone, a potent opioid double receptor agonist, has been gradually applied for pain treatment in clinics.^[5,6]^ In addition, clinical trials have demonstrated that opioids are superior to other classes of drugs in maintaining hemodynamic stability.^[7,8]^ Oxycodone controlled release tablets are considered as novel painkillers with ideal pharmacodynamic and pharmacokinetic effects.^[9–11]^ Meanwhile, the oxycodone injection, with its dual agonist alpha opioids of the μ and κ receptors, has effect on antianxiety, cough relieving and reducing smooth muscle tension, and also has analgesic effect without ceiling effect.^[12]^ As the agonist of κ receptor of oxycodone is more prominent, its analgesic effect on organ pain is better than that of μ-agonist alone.^[13]^

Some studies have shown that oxycodone has beneficial in maintaining hemodynamic stability and postoperative analgesia during laparoscopic surgery. But the effectiveness and feasibility of oxycodone in Da Vinci robot-assisted nephrectomy is not yet clear, and its effect on intraoperative hemodynamics, adverse events, and postoperative pain has not been systematically analyzed. Hence, in this study, we aimed to analyze the feasibility of oxycondone in anesthesia induction and overall maintenance during Da Vinci robot-assisted nephrectomy.

## 2. Materials and Methods

### 2.1. Study population

In this randomized controlled study, patients who were undergoing Da Vinci robot-assisted nephrectomy with general anesthesia were selected. Inclusion criteria were as follows: undergoing nephrectomy, with the age of 18–70 years, BMI 20–25, and American Society of Anesthesiologists I–-II. This study was approved by the ethics committee of Sir Run Run Shaw Hospital. Informed consent form was achieved by all patients or their relatives.

Exclusion criteria were as follows: (1) have allergy to any anesthetics; (2) have underwent heart surgery or suffered from impaired heart function; (3) have respiratory/airway problems, such as respiratory depression or hypoxia, chronic obstructive pulmonary disease, bronchial asthma or other bronchial diseases; (4) have liver diseases or impaired liver function: aspartate aminotransferase, alanine aminotransferase, alkaline phosphatase/normal upper limit >1.5, total bilirubin/normal upper limit >1.5 and γ -glutamyl transpeptidase/normal upper limit >1.5; (5) have renal insufficiency with eGFR <90 mL/min/1.73 m^2^ and creatinine clearance >80–125 mL/min; (6) have contraindications that were indicated in the instructions of oxycodone usage; (7) uncontrolled muscle tension or convulsions; (8) long-term use of painkillers or dependence on other drugs; (9) intracranial hypertension; (10) cognitive dysfunction/communication disorders; (11) pregnant or lactating patients; (12) other conditions that were not suitable for anesthesia with oxycodone.

Withdrawal criteria were as follows: (1) Patients who were not in line with the inclusion criteria, but were mistakenly included. (2) Patients with blood pressure fluctuations of more than 20% after admission, or with recurrent intubation/excessive long-time intubation, or without follow-up records. (3) Patients receiving a combination of drugs that were not specified, especially drugs that had a large impact on clinical outcomes, effectiveness and safety.

### 2.2. Anesthesia management

After admission into operation room and the peripheral venous pathway was opened, patients were given routine monitoring including heart rate (HR) and oxygen saturation (SpO_2_). Then patients underwent radial artery puncture under local anesthesia before induction to monitor the real time systolic blood pressure (SBP), diastolic blood pressure (DBP) and mean arterial blood pressure (MABP). During anesthesia induction, patients were given intravenous injection of oxycodone 0.3 mg/kg or sufentanil 0.3 µg/kg, propofol 1–2 mg/kg, and cis-atracurium 0.2–0.3 mg/kg, followed by tracheal intubation for mechanical ventilation. During anesthesia maintenance period, patients were inhaled sevoflurane 0.8–1.5 MAC, given intermittent intravenous injection of cis-atracurium 0.1 mg/kg and additional oxycodone 0.1 mg/kg if the surgery was longer than 3 hours. The muscle relaxant was suspended 30 minutes before the end of surgery and inhalation of sevoflurane was terminated at the end of surgery. The endotracheal tube was withdrawn after satisfactory recovery of the breath. Patients with a pain score of more than 3 points postoperatively would be given additional nonsteroidal antiinflammatory drugs to relieve from pain.

### 2.3. Observation indexes

Perioperative hemodynamic changes (SBP, DBP, MABP, HR, RR, and SpO_2_) were recorded before induction (T0), after injection of induced drugs (T1), during glottis exposure (T2), immediately after intubation (T3), 1 minute after intubation (T4), 2 minutes after intubation (T5), 3 minutes after intubation (T6), 5 minutes after intubation (T7), 10 minutes after intubation (T8), during skin incision (T9), at the end of suturing skin (T10), during extubation (T11) as well as during hemodynamic fluctuations intraoperatively (T`). Anesthesia time, operation time, pneumoperitoneum time and awakening time were also recorded. Bispectral index (BIS) at T0–T11 as well as T` was evaluated. In addition, frequency of bucking and body movement during anesthesia induction and the entire surgical process were recorded. Postoperative evaluation was performed using numerical rating scale (NRS), facial affective scale (FAS) and visual analogue scale (NVAS) at 1 hour (T’“1), 3 hours (T”“2), 6 hours (T”“3), 12 hours (T”“4), 24 hours (T”“5) and 48 hours (T”“6) after surgery. Patients’ satisfaction with treatment was also assessed using QoR-40. At 24 hours after surgery, patients were evaluated in terms of feelings (ease of breathing, good sleep, good appetite and feeling relaxed), emotions (feeling good and controllable), independence ability (speaking properly, being able to wash and shave, being able to take care of their own appearance, being able to work again, completing their daily activities, writing), patient support (being able to communicate with the medical personnel, being able to communicate with family or friends, getting support from doctors in the hospital, getting support from nurses in the hospital, getting support from family or friends, being able to understand the instructions and advice), bad feelings (nausea, vomiting, retching, anxiety, trembling or convulsions, chills, feeling cold and feeling dizzy), bad emotions (bad dreams, progress, anger, depression, loneliness, difficulty falling asleep), and pain (medium pain, extreme pain, headache, muscle pain, back pain, sore throat, mouth pain), which were scored at 1–5 points: 1 point refers to very bad, and 5 points refers to very good (1: never, 2: 1–3 times, 3: <10 times, 4: >10 times, and 5: always).

### 2.4. Statistical analysis

Measurement data were expressed as mean ± standard deviation (x ± s). Data were compared using nonpaired independent sample K-S test, with an inspection level of α = 0.05. *P* < 0.05 was considered as significant difference.

## 3. Results

A total of 42 patients who successfully underwent Da Vinci robot-assisted nephrectomy were enrolled, including 22 patients in oxycodone group and 20 patients in sufentanil group. Their general information was listed in Table 1. No cough or body movements occurred during the induction of anesthesia and the entire operation. One patient had an NRS score of 5 points at 6 hours and a score of 3 points at 12 hours postoperatively. The patient was then treated with 50 mg flurbiprofen axetil, and the symptoms were relieved. No serious adverse events occurred.

**Table 1:** General information and anesthesia characteristics of patients.

Groups	Oxycodone group	Sufentanil group
Variable	Patients (n = 22)	Patients (n = 20)
Age (yr)	53.91 ± 14.72	48.88 ± 15.33
Body weight (kg)	61.50 ± 10.12	65.59 ± 10.87
Height (cm)	163.77 ± 8.61	168.69 ± 5.70
Surgical site (left/right kidney)	10/12	8/12
SBP (mm Hg)	154.68 ± 30.00	151.00 ± 17.21
DBP (mm Hg)	78.77 ± 9.13	79.82 ± 10.27
MAP (mm Hg)	105.09 ± 15.66	103.52 ± 10.22
HR (/min)	76.41 ± 15.45	77.44 ± 10.22
RR (/min)	76.18 ± 14.87	77.56 ± 10.49
SpO2 (%)	18.14 ± 1.36	16.69 ± 1.74
Anesthesia time (min)	172.55 ± 47.19	168.13 ± 44.30
Operation time (min)	137.09 ± 39.44	144.38 ± 43.05
Pneumoperitoneum time (min)	100.32 ± 40.41	121.25 ± 40.52
Awakening time (min)	31.55 ± 18.76	26.00 ± 14.90
Total amount of oxycodone used in surgery (ml)	19.76 ± 3.68	661.75 ± 155.79
Hospital stay (d)	11.77 ± 3.35	9.31 ± 1.40
Frequency of bucking in induction (times)	0	0
Frequency of bucking intraoperatively (times)	0	0
Frequency of body movement intraoperatively (times)	0	0
Hospital expense (RMB)	76341.43 ± 321.1	73208.5 ± 219.45

### 3.1. Intraoperative hemodynamic changes

**Figure 1.**
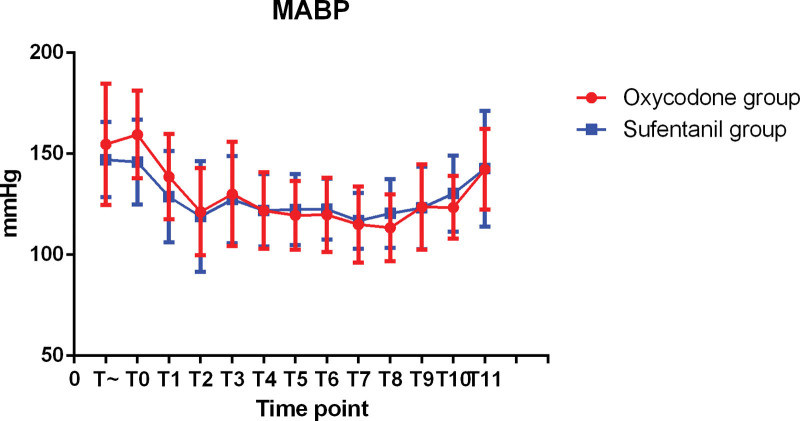
MAP at each intraoperative time point.

**Figure 2.**
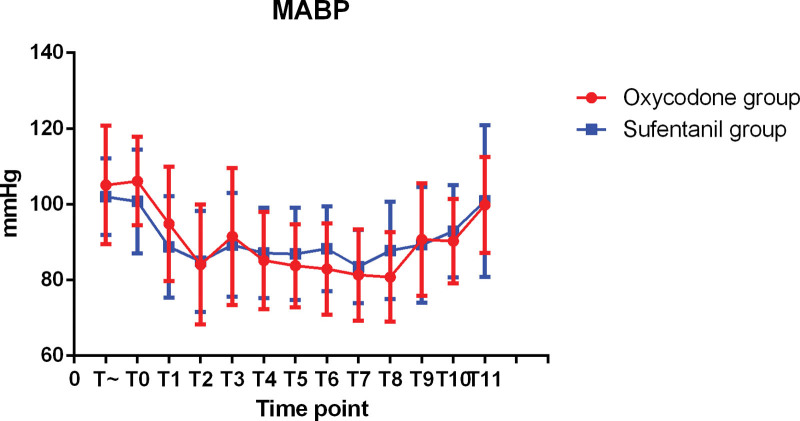
SBP at each intraoperative time point.

**Figure 3.**
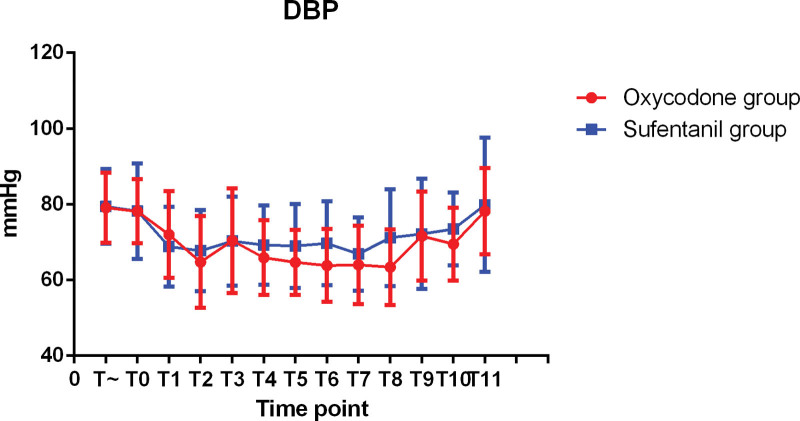
DBP at each intraoperative time point.

**Figure 4.**
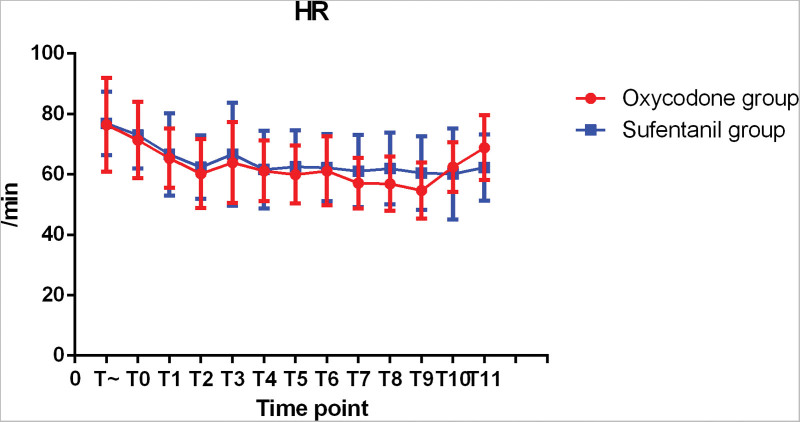
HR at each time point.

The hemodynamic changes during operation were shown in Figures 1–3. There was no significant difference in SBP, MABP, and DBP between 2 groups. The changes of intraoperative heart rate were shown in Figure 4. Heart rate did not show significant difference between 2 groups (*P* > 0.05).

### 3.2. Intraoperative BIS changes

**Figure 5.**
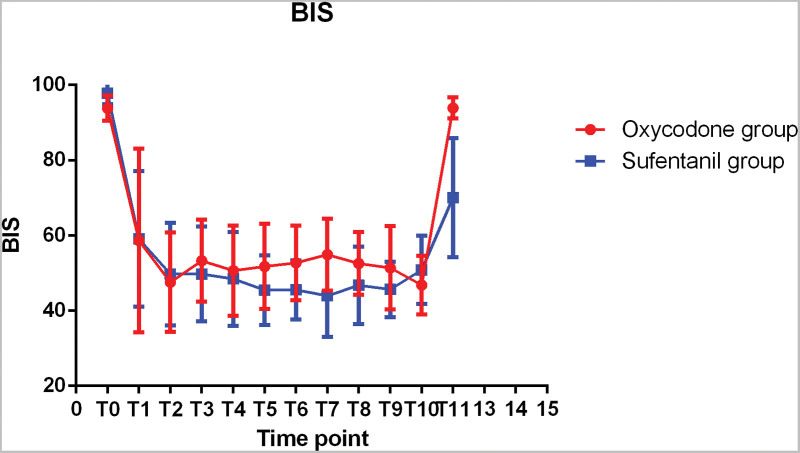
BIS value at each time point.

The changes in BIS during the surgery were displayed in Figure 5. During anesthesia, the BIS value of the 2 groups was between 40 and 60 at each time point, which could achieve the appropriate depth of sedation. The BIS values at T6 were significantly different between the 2 groups (*P* < 0.05). There was no significant difference in BIS between the 2 groups at other time points.

### 3.3. Postoperative NRS and VAS score changes

**Figure 6.**
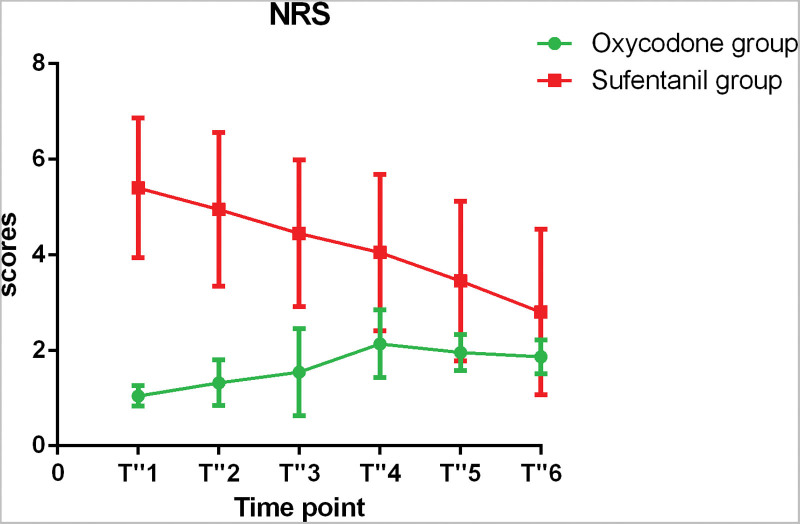
Postoperative NRS at each time point.

The changes of postoperative NRS scores were shown in Figure 6, and the FAS scores and VAS scores were showed in Table 2 and Table 3. The NRS scores at T “1, T” 2, T “3, T” 4 were significantly different between the 2 groups (*P* < 0.05). There was no significant difference in NRS scores at other time points.

**Table 2:** Functional activity scoring system (FAS) in 2 groups

Levels	Oxycodone group			Sufentanil group		
	A	B	C	A	B	C
T’‘1	22	0	0	0	17	3
T’‘2	22	0	0	3	17	0
T’‘3	21	1	0	4	16	0
T’‘4	20	2	0	9	11	0
T’‘5	22	0	0	12	8	0
T’‘6	22	0	0	18	2	0

**Table 3:** Visual analogue scales (NVAS) in 2 groups.

Levels	Oxycodone group				Sufentanil group			
	A	B	C	D	A	B	C	D
T’‘1	22	0	0	0	0	4	14	2
T’‘2	22	0	0	0	0	6	12	2
T’‘3	21	1	0	0	1	11	7	1
T’‘4	20	3	0	0	2	14	4	0
T’‘5	22	0	0	0	4	14	2	0
T’‘6	22	0	0	0	5	14	1	0

### 3.4. Recovery quality (QoR-40) score at postoperative 48 hours

**Figure 7.**
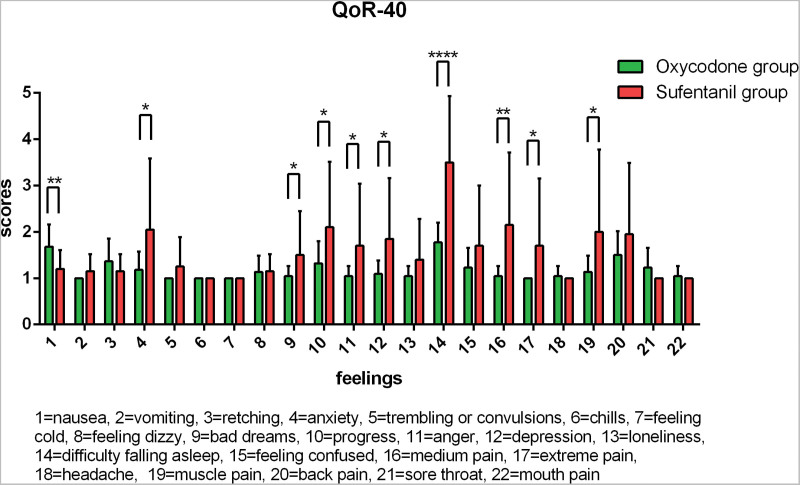
QoR-40 score at postoperative 24 h. Note: 1 = Feelings, 2 = emotions, 3 = independence ability, 4 = patient support. 1-5 points: 1 = very bad, 5 = very good.

The changes of postoperative QoR-40 scores were shown in Figure 7. The scores for feelings, emotions, independence ability and patient support were 4.03 ± 0.4, 3.96 ± 0.38, 4.03 ± 0.31 and 4.99 ± 0.04 in oxycodone group, while the scores were 2.78 ± 1.78, 2.83 ± 1.68, 1.68 ± 1.47, 4.77 ± 0.59 in sufentanil group respectively. Especially in feelings, emotions and independence ability, the scores in oxycodone group were significant higher than those in sufentanil group (*P* < 0.05). In terms of postoperative adverse symptoms, there were significant differences between the 2 groups in retching, difficulty falling asleep, moderate pain and sore throat (*P* < 0.05). And the other adverse reactions showed no significant difference between the 2 groups.

## 4. Discussion

Partial nephrectomy with Da Vinci robot remains a major trend in future, which puts forwarded higher requirements for intraoperative anesthesia management.^[14]^ It is important to maintain the stability of hemodynamics to reduce the effect of intraoperative stress damage on clinical prognosis, and to provide good analgesic measures to improve satisfaction and fast recovery of patients. In this randomized controlled study, we firstly demonstrated that anesthesia management using oxycodone during Da Vinci robot-assisted nephrectomy can achieve a promising advantage characterized by stable hemodynamics, less adverse reactions and good postoperative analgesia. Our work indicates that using oxycodone may contribute to prevent adverse events and relieve analgesia although further work is needed to valid oxycodone for it to be a potential and ubiquitous integral part of anesthesia use for Da Vinci robot surgical patients.

Compared with traditional opioids, oxycodone has characteristics of rapid onset, long elimination half-life, and low adverse reaction, while does not have similar effect of immunosuppression as morphine and fentanyl, and is unlikely to cause histamine release and bradycardia, and inhibit parasympathetic nerves.^[15]^ Like morphine, oxycodone is a m-opioid receptor agonist with a significantly different pharmacokinetic profile compared with morphine. It seems that oxycodone is able to compensate its lower binding affinity for the m-opioid receptor compared with that of morphine by active transport to the central nervous system. Thus, it can be used as an opioid analgesic during induction of anesthesia.^[16]^ Several studies have demonstrated the application of oxycodone in perioperative period. In a previous study by Piirainen et al, oxycodone, used for anesthesia induction with a dose of 0.20 mg/kg and titrated postoperatively with a dose of 0.00–0.33 mg/kg during laparoscopic cholecystectomy (LC), showed a low incidence of adverse response.^[17]^ Another study also found that the use of 0.08 mg/kg oxycodone during the perioperative period of LC significantly inhibited postoperative pain, while did not increase the adverse events.^[18]^ Park et al found that oxycodone with the dose of 0.14 mg/kg and 0.20 mg/kg could significantly reduce the variation amplitude of blood pressure and heart rate during anesthesia induction compared with fentanyl in nephrectomy.^[19]^ And an induction dose of 1.0 mg/kg and a maintenance dose of 0.5 mg/kg/h with oxycodone could maintain hemodynamic stability throughout the anesthesia period.^[16]^ A domestic study comprehensively evaluated the hemodynamics, adverse events, and pain scores for LC with multi-step doses of oxycodone, suggesting the appropriate dose for oxycodone induction was 0.30 mg/kg.^[20]^

With regard to the changes in BIS during operation, there was no significant difference between the 2 groups. BIS value in oxycodone group was a little higher than that in sufentanil group at 3–5 minutes after intubation, which may be related to the dosage of oxycodone, individual differences of patients and other environmental factors. After operation, the BIS score in oxycodone group was significantly higher than that in sufentanil group, indicating a better recovery after anesthesia.

In the other hand, we found that patients receiving oxycodone experienced mild postoperative pain. The pain was aggravated slowly after surgery till 12 hours postoperatively, and peaked with a maximum of 2.18 ± 0.73. The pain was mild and did not affect the sleep of the patient. Then pain was slowly relieved from 12 hours to 48 hours postoperatively, when the score was decreased to 1.82 ± 0.39. However, in sufentanil group, at 1 hour after operation, the score reached highest and then decreased slowly with time. At 12 hours, the score was still 3.44 ± 1.88, which was significantly higher than that of oxycodone group. At 48 hours, it dropped to 1.67 ± 1.80, which was at the same level as that of oxycodone group. From the pharmacokinetics point of view, the half-life of oxycodone is up to 3.5 hours, resulting in a relatively stable effect on postoperative analgesia. Yang et al^[21]^ found that the postoperative NRS scores in 41 laparoscopic nephrectomy patients who were given analgesic pump was 2.15 ± 1.59, which was similar to the postoperative NRS scores in the present study. We also find that in the case of normal postoperative analgesic doses, the median analgesic effect of oxycodone and analgesic pump are similar, but oxycodone has better analgesic properties in terms of pain stability and shows a small difference between different patients.

In terms of VAS score for adverse events, in sufentanial group, there were 15 patients with score B and 1 patient with score C at 1 hour after surgery. Then there were 7 patients with score B at 24 hours. At 48 hours, only 2 patients had score B. In oxycodone group, only 3 patients were rated as B at 12 hours postoperatively, indicating good analgesic effect. QoR-40 score analysis for postoperative recovery quality show that the scores for feelings, emotions, independence ability and patient support in oxycodone group were 4.03 ± 0.4, 3.96 ± 0.38, 4.03 ± 0.31, and 4.99 ± 0.04, indicating most patients have good emotion and could take care of themselves within a certain range and perform normal communication. As for the postoperative adverse reactions, the incidence of retching and sore throat in oxycodone group was slightly higher, while the incidence of difficulty in falling asleep and moderate pain was lower than that in sufentanil group, suggesting oxycodone has obvious advantages in analgesia after surgery. In addition to the possibility of side effects caused by anesthetic drugs, adverse events might be related to the surgical process, postoperative mental stress and postoperative pain. With reference to the recommendations in BISGAARD,^[21]^ dexamethasone can be used as a prophylactic before surgery. Furthermore, postoperative education, early postoperative recovery and other perioperative treatments can also effectively reduce the incidence of adverse reactions after surgery.

It should be noted that oxycodone is also a type of opioid agonist, with the possibility of abstinence reaction after discontinuation, but its abuse risk is much lower than that of other opioid receptor agonists.^[15]^ From the postoperative response point of view, the incidence of postoperative tension was 6/17, anxiety was 4/17 and convulsions was 0/17, which may be related to the abstinence reaction after discontinuation. Liu et al^[22]^ found that on the 4^th^ day of 5 mg/kg/day oxycodone injection intraperitoneally, the spontaneous activity of Wistar rats was significantly increased compared with that on the first day. In addition, Wang et al^[23]^ found that cancer pain patients who had abstinence reaction after oral administration of oxycodone hydrochloride prolonged-release tablets generally received treatment for more than 3 weeks, and often manifested with palpitations, insomnia and excitement. Abstinence symptoms can be successfully avoided by reducing the dose or by even discontinuing the medication. In the present study, patients showed no obvious specific abstinence symptoms after discontinuation of oxycodone, which might be associated with a relatively small dose of medication and relatively short medication duration. However, the specific dosage of oxycodone and the relationship between the usage time and the abstinence symptoms are needed further investigation.

There are some factors that can influence the outcome of the study. First, the study was conducted in 1 hospital center and multiple centers can be done in future to increase the population involved. Second, the participants were most in young population group and it is suggested to include more elderly patients in next study plan. However, this study is not without limitations. The sample size of the study is relative small. Further study is needed to verify these findings by increasing the sample size.

## 5. Conclusions

In summary, the application of oxycodone in induction period and overall management during Da Vinci robot-assisted nephrectomy is characterized by stable hemodynamics, less adverse reactions and good postoperative analgesia.

### Author contributions

HHW conceived and coordinated the study, designed, performed and analyzed the experiments, wrote the paper. YLQ, QZ, YJC carried out the data collection, data analysis, and revised the paper. LM designed the study, carried out the data analysis and revised the paper. All authors reviewed the results and approved the final version of the manuscript.
